# Carnosine suppresses human colorectal cancer cell proliferation by inducing necroptosis and autophagy and reducing angiogenesis

**DOI:** 10.3892/ol.2021.13162

**Published:** 2021-12-09

**Authors:** Shu-Ling Hsieh, Jia-Huei Li, Cheng-Di Dong, Chiu-Wen Chen, Chih-Chung Wu

**Affiliations:** 1 Department of Seafood Science, National Kaohsiung University of Science and Technology, Kaohsiung 81157, Taiwan, R.O.C.; 2 Marine Environmental Engineering, National Kaohsiung University of Science and Technology, Kaohsiung 81157, Taiwan, R.O.C.; 3 Department of Food and Nutrition, Providence University, Taichung 43301, Taiwan, R.O.C.

**Keywords:** carnosine, cell proliferation, necroptosis, apoptosis, autophagy, angiogenesis

## Abstract

Carnosine (β-alanyl-L-histidine) is found in beef and fish. The present study aimed to investigate the effects of carnosine on the cell proliferation of human colorectal cancer cells. After human colorectal cancer HCT-116 cells were treated carnosine for 72 or 96 h, the cell proliferation, apoptosis, autophagy, necroptosis, angiogenesis and the expression of related regulatory molecules were detected using MTT assays, fluorescence image analysis and RT-qPCR in this study. Treatment of HCT-116 cells with 5, 10 or 15 mM carnosine for 72 or 96 h significantly decreased cell viability (P<0.05). The mRNA expression of β-catenin and transcription factor 4 (Tcf-4) was significantly reduced by 15–23% and 11–80%, respectively (P<0.05). When HCT-116 cells were treated with 15 mM carnosine, the mRNA levels of 1A/1B-light chain 3 and phosphatidylinositol 3-kinase were significantly increased by 235% and 249%, respectively (P<0.05). The mRNA level of Beclin-1 and autophagy levels were significantly increased by 137–141% in HCT-116 cells treated with 5, 10 or 15 mM carnosine (P<0.05). Carnosine (15 mM) also increased reactive oxygen species levels and mixed lineage kinase domain-like protein mRNA expression and depleted ATP levels (P<0.05). The angiogenesis-regulating molecules vascular endothelial growth factor, epidermal growth factor receptor and hypoxia-inducible factor 1-α were all significantly decreased by 10 or 15 mM carnosine treatment. These results showed that carnosine could suppress human colorectal cell proliferation by reducing β-catenin/Tcf-4 signaling, inducing autophagy and necroptosis and inhibiting angiogenesis. It was demonstrated that carnosine is a potential compound from dietary food for the future clinical treatment and/or prevention of colorectal cancer.

## Introduction

Carnosine (β-alanyl-L-histidine) is a natural dipeptide that is found in muscle and brain tissue, especially in lean beef, fish and chicken (1). Previous studies have shown that carnosine has a number of physiological effects, including antiaging effects (2), cerebral protection (3), antioxidation (4), inhibiting inflammation (5), reducing Parkinson's disease (6) and inhibiting metastasis (7,8). Carnosine has been shown to inhibit the proliferation of human gastric carcinoma cells by regulating Akt and mammalian target of rapamycin (mTOR) signaling (9). In addition to the physiological effects of carnosine itself, there are various physiological and regulatory effects that are induced by the metabolites of carnosine. During methylation, carnosine will be reacted to anserine and ophidine. These reactions lead to methylhistidine formation, which is an important indicator of muscle breakdown (10). However, the hydrolysis of carnosine produces histidine and β-alanine. β-alanine is involved in the synthesis of CoA, nucleic acids and histidine, the decarboxylation of which yields histamine (11). Carnosine reduces apoptosis in murine podocytes by reducing Bcl-2-associated X protein (Bax) and increasing B-cell lymphoma-2 (Bcl-2) mRNA levels (12). Lee *et al* (13) also show that carnosine can induce apoptosis and cell cycle arrest to lead to reduced cell viability in human colorectal HCT-116 cells. However, the investigation of carnosine-mediated suppression of cell proliferation and induction of cell growth in human colorectal cells is remains an important issue.

Worldwide, colorectal cancer (CRC) is the third most diagnosed cancer and the fourth leading cause of mortality (14). In addition, the incidence of CRC was 10.2% and the mortality was 9.0% in 2018 according to global cancer statistics (15). Instead of clinical cancer treatments, such as surgery, chemotherapy and radiotherapy, chemoprevention by active compounds in food to reduce or inhibit cancer cell proliferation could be a good strategy for reducing cancer-related damage to human health. Between the various forms of cell death such as necrosis, apoptosis, autophagy and necroptosis, a balance exists between normal cell proliferation and growth, abnormal cancer cell proliferation and programmed cell death induction (16). Wnt/β-catenin/transcription factor 4 (Tcf-4) signaling activation serves an important role in regulating cell proliferation (17). This transcription process can modify the cell cycle distribution and cell proliferation (17). Apoptosis serves a major role in the regulation of carcinogenesis (18). Apoptosis is controlled by a large number of genes, such as the Bcl-2 family genes and cysteine proteases and is regulated by signaling pathways (19). Autophagy is triggered by various types of intracellular stress, including DNA damage and low nutrient levels (20). The hyperactivation of autophagy can lead to autophagic cell death. Akt/mTOR/1A/1B-light chain 3 (LC3) signaling is an important pathway for autophagy induction (21). Recently, necroptosis was demonstrated to be a new form of programmed cell death that differs from apoptosis but is similar to necrosis (22). Necroptosis is involved in specific physiological and pathological processes (22). Receptor interaction protein 3 (RIP3) and mixed lineage kinase domain-like protein (MLKL) are both required for the activation of necroptosis (23). Excessive reactive oxygen species (ROS) production not only acts as a signal to stimulate apoptosis and DNA damage but is also associated with necroptosis (24). In addition, angiogenesis, which is involved in tumorigenesis, is regulated by specific molecules, such as vascular endothelial growth factor (VEGF), epidermal growth factor receptor (EGFR) and hypoxia-inducible factor 1-α (HIF-α) (25). These molecules are also important for tumorigenesis (26). Proliferation and cell death pathways are involved in inhibiting tumorigenesis (26). The present study investigated the effects of carnosine on reducing cell proliferation and inhibiting tumorigenesis as a potential chemoprevention strategy and aimed to investigate the effects of carnosine on cell proliferation, apoptosis, autophagy, necroptosis and angiogenesis in human CRC cells.

## Materials and methods

### Reagents

Carnosine {2ST-2-[(3-amino-1-oxopropyl) amino]-3-(3H-imidazol-4-yl)} propanoic acid; β-alanyl-L-histidine was purchased from Sigma-Aldrich (Merck KGaA).

### Cell culture and carnosine treatment

The human colon carcinoma cell line HCT-116 was purchased from the Bioresource Collection and Research Center. HCT-116 cells (passages 43–65) were maintained in DMEM (Gibco; Thermo Fisher Scientific, Inc.) supplemented with 10% FBS (Gibco; Thermo Fisher Scientific, Inc.) and 1% penicillin/streptomycin (Gibco; Thermo Fisher Scientific, Inc.) at 37°C in a 5% CO_2_ humidified atmosphere.

In the present study, 1×10^5^ HCT-116 cells per 30-mm culture plate were used for mRNA expression analysis and 5×10^5^ HCT-116 cells per 60-mm culture plate were used to determine cell viability, apoptosis and autophagy percentages and ROS and ATP levels. After a series of preliminary tests, the optimum experimental concentration of carnosine was confirmed to be 0.5–15 mM. To determine the effects of carnosine on cell viability, various cell death-regulating molecules were analyzed in cultured cells that were treated with 0.5, 1, 5, 10 or 15 mM carnosine for 72 or 96 h. Carnosine was dissolved in sterilized H_2_O and cells that were treated with only H_2_O were used as a control group.

### Cell viability and morphological analysis

The 3-(4,5-dimethyl-2-yl)-2,5-diphenyl tetrazolium bromide (MTT; Sigma-Aldrich; Merck KGaA) assay and morphological examination were used to assess cell viability. The MTT assay was performed as described by Denizot and Lang (1986) (27). HCT-116 cells were treated with 0.5, 1, 5, 10 or 15 mM carnosine for 72 or 96 h; MTT reagent (5 mg/ml) was then added and the cells were incubated for 3 h at 37°C, followed by extraction with 1 ml of isopropanol after the cells were washed with cold phosphate-buffered saline (PBS; 3.2 mM Na_2_HPO_4_, 0.5 mM KH_2_PO_4_, 1.3 mM KCl, 135 mM NaCl, pH 7.4). Cell viability was determined by measuring the optical density at 570 nm using a Microplate Biokinetics Reader (BioTek Instruments, Inc.). A phase-contrast inverted fluorescence microscope was used to determine morphological changes (Olympus IX 51; Olympus Corporation).

### Analysis of apoptosis and autophagy percentages

To determine the apoptosis percentage, an Annexin V Assay using the NucleoCounter^®^ NC-3000 System (ChemoMetec Inc.) was used. HCT-116 cells were treated with 0.5, 1, 5, 10 or 15 mM carnosine for 96 h and then washed with cold PBS twice. HCT-116 cells were suspended in 100 µl of Annexin V binding buffer with 2 µl of Annexin V-CF488A conjugate (FITC-labeled Annexin V; staining early stage apoptoic cells) and 2 µl of Solution 15 (10 µg/ml Hoechst 33342 to stain the total population). Then, the cells were incubated for 15 min at 37°C and centrifuged at 400 × g for 5 min at 4°C. After the supernatant was removed, the cell pellets were resuspended in 300 µl of Annexin V binding buffer and centrifuged at 400 × g for 5 min at 4°C. The cell pellets were resuspended in 100 µl of Annexin V binding buffer and 2 µl of Solution 16 (500 µg/ml propidium iodide; staining late apoptotic cells) was added. Using 8-chamber NC-Slides A8, analysis was performed via NucleoCounter^®^ NC-3000 fluorescence image cytometer (ChemoMetec Inc.).

HCT-116 cell autophagy in response to carnosine treatment was analyzed by a CYTO-ID^®^ autophagy detection kit (cat. no. ENZ-51031; Enzo Life Sciences, Inc.). HCT-116 cells were treated with 0.5, 1, 5, 10 or 15 mM carnosine at 37°C in a 5% CO_2_ incubator for 96 h and then washed with PBS at room temperature twice. Then, the cells were centrifuged at 400 × g for 5 min at room temperature. After the supernatant was removed, the cell pellets were resuspended in 200 µl PBS at room temperature for 20 min and 0.4 µl Cyto-ID Green stain solution was added for 5 min of staining at room temperature and then 0.2 µl Hoechst 33342 stain solution was added for 20 min of staining at room temperature. The number of autophagic vacuoles was measured after the cells were harvested and stained with Cyto-ID Green fluorescent dyes (excitation, ~480 nm; emission, ~530). Using 2-chamber NC-Slides A2, analysis was performed using a NucleoCounter^®^ NC-3000 fluorescence image cytometer (ChemoMetec Inc.).

### Reverse transcription-quantitative (RT-q)PCR analysis of the mRNA expression of autophagy-, necroptosis- and angiogenesis-associated genes

A total of ~5×10^5^ HCT-116 cells/30-mm plate were incubated in the absence or presence of 0.5, 1, 5, 10 or 15 mM carnosine for 96 h with 5% CO_2_ at 37°C and total RNA was isolated using a modified version of the method described by Chomczynski and Sacchi (1987) (28). The cells were added to 1 ml TRIzol reagent (Sigma-Aldrich; Merck KGaA) and mixed thoroughly, and then 200 µl chloroform was added and mixed thoroughly. Cooling on ice for 5 min was followed by centrifugation at 16,200 × g for 15 min at 4°C. To precipitate RNA, the aqueous phase (500 µl) was transferred to a fresh tube and mixed with 500 µl isopropanol cooled on ice for 5 min, followed by centrifugation at 16,200 × g for 15 min at 4°C. The RNA pellet was washed with 75% (v/v) ethanol and the supernatant was removed. The pellet was air-dried at room temperature and then resuspended in 30–50 µl DEPC-treated water and stored at −80°C until use. The total RNA purity was determined by measuring the absorbance on an Epoch microplate spectrophotometer (BioTek Instruments, Inc.). The absorption ratios (260/280 nm) of all RNA samples were >2, and this indicated that the total RNA was of high quality. The RNA samples were stored at −80°C until use. Total RNA (3 µg) was used for RT, which was performed using Superscript III reverse transcriptase (Thermo Fisher Scientific, Inc.) and oligo d(T)21 as a primer, and the reaction conditions used were as recommended by the manufacturer. Real-time PCR primers for β-catenin, Tcf-4, Bax, Bcl-2, Caspase-3, Caspase-8, poly(ADP-ribose) polymerase (PARP), MLKL, Beclin-1, LC3, PI3K III, VEGF, EGFR and HIF-α were prepared and designed as previously described (7). The thermal profile for the real-time quantitative PCR was 95°C for 2 min and 95°C for 10 min, followed by 40 cycles of 95°C for 15 sec and 60°C for 1 min, according to the manufacturer's protocol. Gene expression is presented as the fold change relative to the β-actin level, which was calculated as 2^−ΔΔCq^ as previously described (29) and is shown in Table I. In addition, melting curve analysis was performed to assure the specificity of the PCR products in this experiment. The results were obtained from three independent experiments (n=3).

### Statistical analysis

All experimental data were analyzed using SPSS statistical analysis software for Windows, version 20.0 (IBM Corp.). One-way analysis of variance and Tukey's post hoc test were used to evaluate the significance of differences between two mean values. P<0.05 was considered to indicate a statistically significant difference.

## Results

### Carnosine suppresses the proliferation of HCT-116 cells

**Figure 1.**
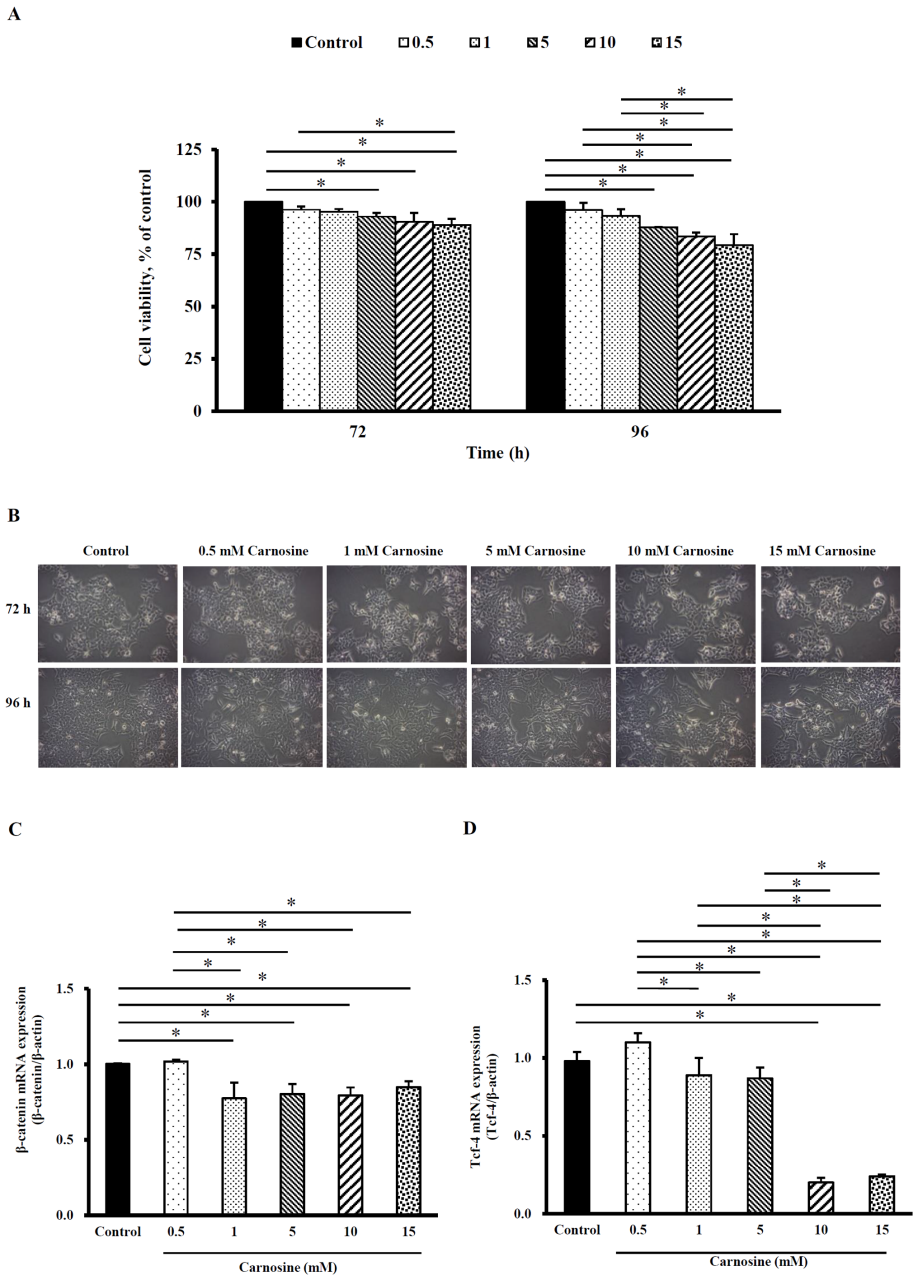
Effect of carnosine on the viability and proliferation-related molecule expression of HCT-116 cells. HCT-116 cells (5×10^5^ cells per 60-mm plate for the cell viability and morphology assays and 1×10^5^ cells per 30-mm plate for the mRNA expression analysis) were treated with 0.5, 1, 5, 10 or 15 mM carnosine for 72 or 96 h. The control group was treated with sterilized H_2_O. (A) Cell viability, (B) morphological changes (magnification, ×100), (C) β-catenin mRNA expression and (D) Tcf-4 mRNA expression were examined. (A and B) cell viability was tested at 72 and 96 h; (C and D) expression levels were detected at 96 h. The values are presented as the mean ± SD (n=3-5). *P<0.05. Tcf-4, transcription factor 4.

When HCT-116 cells were treated with 5, 10 or 15 mM carnosine for 72 h, the viability was 93.0±1.8%, 90.0±4.3% and 89.0±2.9%, respectively. The viability of HCT-116 cells was 87.9±0.2%, 83.5±1.9% and 79.4±5.2% following treatment with 5, 10 or 15 mM carnosine for 96 h, respectively. The viability was significantly lower than that of the control group (100%) (P<0.05) after 72 and 96 h of incubation (Fig. 1A). However, cell morphology was examined by inverted microscopy and cell number in each of the various carnosine treatment groups was similar to that in the control group (Fig. 1B). Therefore, the employed carnosine concentrations were used in all tests in this study. Fig. 1C shows that the β-catenin mRNA levels were significantly reduced in HCT-116 cells treated with 1, 5, 10 or 15 mM carnosine for 96 h by 15–23% compared with those in the control group (P<0.05). When HCT-116 cells were treated with 1, 5, 10 or 15 mM carnosine, the Tcf-4 mRNA levels were significantly reduced by 11–76% compared with those of the control group (P<0.05) (Fig. 1D). These results demonstrate that carnosine can reduce cell proliferation and the carnosine-induced reduction in β-catenin/Tcf-4 signaling activation may be involved in HCT-116 cell proliferation.

### Carnosine did not induce apoptosis in HCT116 cells

**Figure 2.**
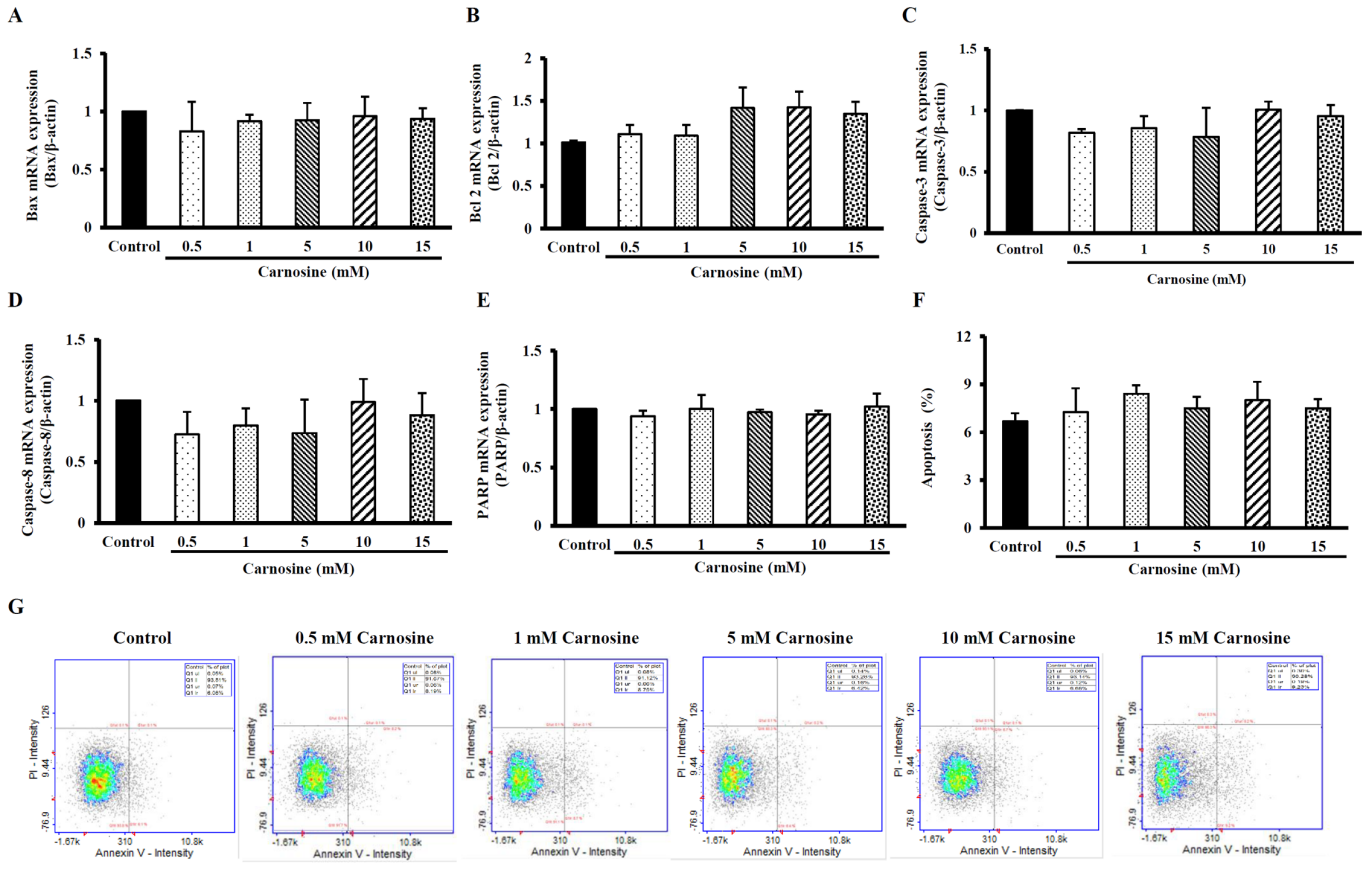
Effect of carnosine on apoptosis levels and the mRNA expression of apoptosis-related molecules in HCT-116 cells. HCT-116 cells (1×10^5^ cells per 30-mm plate for the mRNA expression analysis and 5×10^5^ cells per 60-mm plate for the apoptosis assay) were treated with 0.5, 1, 5, 10 or 15 mM carnosine for 96 h. The control group was treated with sterilized. The mRNA expression of (A) Bax, (B) Bcl-2, (C) Caspase-3, (D) Caspase-8, (E) PARP, (F) the levels of apoptosis and (G) apoptosis via FITC-labeled Annexin V staining of HCT-116 cells were examined. The values are presented as the mean ± SD (n=3-5). Bax, Bcl-2-associated X protein; Bcl-2, B-cell lymphoma-2; PARP, poly(ADP-ribose) polymerase.

RT-qPCR analysis showed that the treatment of HCT-116 cells with 5, 10 or 15 mM carnosine did not affect the mRNA expression of Bax, Bcl-2, Caspase-3, Caspase-8, or PARP (Fig. 2 A-E). In addition, after HCT-116 cells were incubated with 5, 10 or 15 mM carnosine, the apoptosis percentage was no different from that of the control group (Fig. 2F and G). Carnosine (1–15 mM) did not induce apoptosis in HCT-116 cells even after 96 h of treatment.

### Carnosine induces autophagy in HCT116 cells

**Figure 3.**
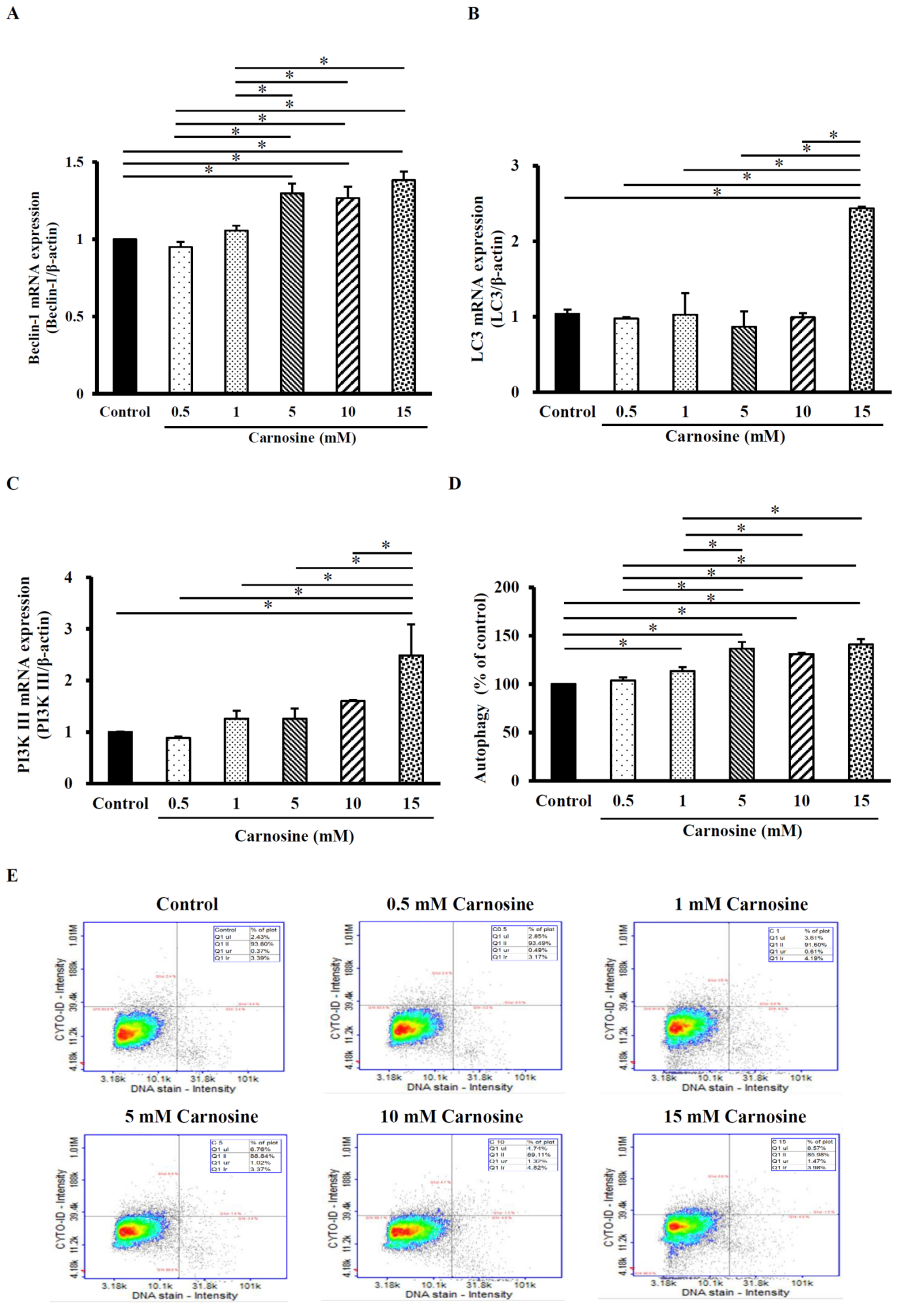
Effect of carnosine on autophagy levels and the mRNA expression of autophagy-related molecules in HCT-116 cells. HCT-116 cells (1×10^5^ cells per 30-mm plate for the mRNA expression analysis and 5×10^5^ cells per 60-mm plate for the autophagy assay) were treated with 0.5, 1, 5, 10 or 15 mM carnosine for 96 h. The control group was treated with sterilized H_2_O. The mRNA expression of (A) Beclin-1, (B) LC3, (C) PI3K III, (D) autophagy via Cyto-ID Green staining of HCT-116 cells and (E) autophagy levels were examined. The values are presented as the mean ± SD (n=3-5). *P<0.05. LC3, 1A/1B-light chain 3; PI3K, phosphatidylinositol 3-kinase.

Beclin-1 mRNA expression in HCT-116 cells was significantly increased by 6–38% after 5, 10 or 15 mM carnosine treatment for 96 h compared with those in the control group (P<0.05; Fig. 3A). The LC3 mRNA expression levels were also significantly increased by 144% after the cells were treated with 15 mM carnosine for 96 h compared with those in the control group (Fig. 3B) and PI3K III mRNA levels were significantly increased by 149% compared with those of the control group (P<0.05; Fig. 3C). In addition, the formation of autophagosomes (stained by LYSO-ID1 Green dye) increased after carnosine treatment compared with the control group (Fig. 3D). Autophagy was significantly increased by 13–41% after 1, 5, 10 or 15 mM carnosine treatment for 96 h (P<0.05; Fig. 3D and E). These results showed that 15 mM carnosine can induce autophagy by regulating the indicated regulatory molecules. However, 0.5 mM carnosine did not exert any effect and was equal to control.

### Carnosine induces necroptosis in HCT116 cells

**Figure 4.**
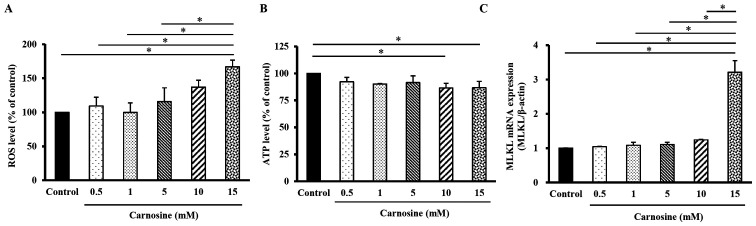
Effect of carnosine on the mRNA expression of necroptosis-related molecules in HCT-116 cells. HCT-116 cells (1×10^5^ cells per 30-mm plate for the mRNA expression analysis or 5×10^5^ cells per 60-mm plate for the ROS and ATP level assays) were treated with 0.5, 1, 5, 10 or 15 mM carnosine for 96 h. The control group was treated with sterilized H_2_O. the levels of (A) ROS, (B) ATP and (C) MLKL mRNA were examined. The values are presented as the mean ± SD (n=3-5). *P<0.05. ROS, reactive oxygen species; MLKL, mixed lineage kinase domain like pseudokinase.

As shown in Fig. 4A, the levels of ROS in HCT116 cells treated with 15 mM carnosine for 96 h were significantly increased by 67% compared with those in the control group (100%; P<0.05). The ATP levels in HCT-116 cells treated with 10 or 15 mM carnosine were both significantly decreased by 13% compared with those in the control group (100%; P<0.05; Fig. 4B). The MLKL mRNA expression level in HCT-116 cells treated with 15 mM carnosine was significantly increased by 221% compared with that in the control group (P<0.05; Fig. 4C). These results showed that 10–15 mM carnosine could induce necroptosis in HCT-116 cells.

### Carnosine suppresses angiogenesis in HCT116 cells

**Figure 5.**
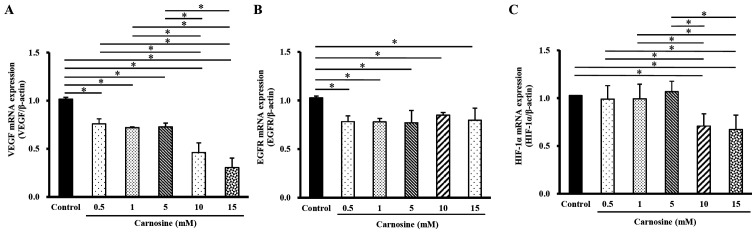
Effect of carnosine on the mRNA expression of angiogenesis-related molecules in HCT-116 cells. HCT-116 cells (1×10^5^ cells per 30-mm plate for the mRNA expression analysis) were treated with 0.5, 1, 5, 10 or 15 mM carnosine for 96 h. The control group was treated with sterilized H_2_O. The mRNA expression of (A) VEGF, (B) EGFR and (C) HIF-α was examined. The values are presented as the mean ± SD (n=3-5). *P<0.05. VEGF, vascular endothelial growth factor; EGFR, epidermal growth factor receptor; HIF-α, hypoxia-inducible factor 1-alpha.

The mRNA levels of VEGF in HCT-116 cells treated with 10 or 15 mM carnosine for 96 h were significantly lower (24% or 70%, respectively) than the control group (100%; P<0.05; Fig. 5A), while EGFR mRNA expression significantly decreased by 15–22% following treatment with 0.5, 1, 5, 10 or 15 mM carnosine for 96 h (P<0.05; Fig. 5B). However, the mRNA levels of HIF-α in cells exposed to 10 and 15 mM carnosine for 96 h significantly decreased by 29% and 33%, respectively, compared with those in the control group (Fig. 5C). Carnosine significantly reduced HCT-116 cell angiogenesis by reducing these angiogenesis-regulating molecules.

## Discussion

In the present study, carnosine significantly suppressed the proliferation and viability of HCT-116 human CRC cells by inducing necroptosis and autophagy and inhibiting angiogenesis. In the current study, the mRNA expression of β-catenin and Tcf-4, two key molecules in cell proliferation-associated signaling, was decreased after HCT-116 cells were treated with carnosine. Carnosine could increase the mRNA expression levels of Beclin-1 and PI3K and reduced LC-3 mRNA expression, leading to autophagy in HCT-116 cells. When HCT-116 cells were treated with carnosine, ATP levels were significantly decreased and ROS levels and MLKL mRNA expression were significantly increased. Carnosine could induce necroptosis to decrease the viability of HCT-116 cells. In addition, VEGF, EGFR and HIF-α mRNA levels were significantly reduced after HCT-116 cells were treated with carnosine. Carnosine could reduce angiogenesis in HCT-116 cells by reducing these angiogenesis-regulatory molecules. These results showed that carnosine can reduce colorectal cell proliferation and induce cancer cell death. However, the molecular regulatory mechanism and gene expression modifications require further study.

In the clinic, there are several chemotherapeutic drugs that have been reported to participate in cell proliferation, autophagy, apoptosis and angiogenesis. For example, 5-fluorouracil can regulate cell proliferation by reducing Wnt/β-catenin signaling (30), temozolomide and sorafenib both can induce autophagy by inducing LC3 expression (31,32), dabrafenib can induce necroptosis by regulating MLKL expression (33) and lenvatinib can exhibit anti-angiogenesis effects by reducing VEGF expression (34). In the present study, carnosine could reduce cell proliferation, induce autophagy and necroptosis and suppress angiogenesis. It has a marked potential for cancer prevention and therapy.

The proliferation of normal cells includes cell growth and division to replace lost cells (35). The cell cycle serves a central role in cell growth and proliferation. Abnormal regulation of the cell cycle can lead to the over proliferation of cells and an accumulation of abnormal numbers of cells (35). However, apoptosis is usually an important pathway for the removal of excess and impaired cells in normal tissue and organs (36). Investigating how to suppress abnormal cell proliferation and cell growth by active compounds is an important issue in cancer proliferation and cell growth (37).

The present study showed that the treatment of HCT-116 cells with 0.5–15 mM carnosine for 96 h did not induce apoptosis. The results showed that the mRNA levels of the proapoptotic factor Bax and the apoptotic factor Bcl-2 in HCT-116 cells were not affected by treatment with carnosine for 96 h. In addition, there were similar results showing that the intrinsic apoptotic pathway marker Caspase-3, the extrinsic apoptotic pathway marker Caspase-8 and PARP in HCT-116 cells were not affected by carnosine treatment. However, Lee *et al* (13) showed that 200 mM carnosine can induce apoptosis in HCT-116 cells after 24 h by increasing Bax, Caspase-3 and cyclin D1 protein levels. Carnosine (200 mM) can also induce apoptosis in SGC-7901 and MKN45 human gastric carcinoma cells by reducing Bcl-2 and increasing Bax and PARP protein expression (9). In addition, Shi and Zhang (38) (2011) showed that 20 mM carnosine could protect human umbilical vein endothelial cells (HUVECs) from high glucose-induced apoptosis. Carnosine (5–20 mM) can also protect murine podocytes from high glucose-induced apoptosis by reducing Bax and Caspase-3 levels (12). Tiwari (36) (2011) showed that exogenous and endogenous compounds induce, suppress, or exhibit no effects on apoptosis and the cellular dose response and kinetics must be considered. The dose and treatment times of compounds determine not only the sensitivity and tolerance time but also the form of cell death (36). These aforementioned studies (9,12,13,38) have shown that carnosine regulates apoptosis and the dose-response relationship between apoptosis and the treatment agent. These results differ from those of the present study regarding apoptosis induction. Different experimental models, carnosine doses and treatment times may cause differences in apoptosis induction.

Although carnosine did not induce apoptosis, it did block the reduction in β-catenin and Tcf-4 activation by reducing β-catenin and Tcf-4 expression in the present study. Wnt/β-catenin/Tcf-4 signaling is a major transcriptional regulator of c-myc, cyclin D1 and VEGF, which are important regulators of the cell cycle, cell proliferation and angiogenesis (39,40). In addition, Sebio *et al* (41) showed aberrant Wnt/β-catenin signaling is a characteristic feature of colorectal cancer (CRC). In the present study, carnosine reduced β-catenin and Tcf-4 expression, which may be important in reducing the proliferation of HCT-116 cells. Previous studies (39,40,42,43) have shown that when HCT-116 cells were treated with celecoxib, a selective cyclooxygenase-2 inhibitor, Tcf-4 expression was significantly inhibited and Wnt/β-catenin and Tcf-4 expression was blocked, reducing human colon cancer cell proliferation (42). Quercetin can also reduce SW480 cell proliferation through the downregulation of Wnt/β-catenin/Tcf-4 expression (43). In cancer prevention and therapy, the regulation of β-catenin/Tcf4 signaling to reduce abnormal cell proliferation with active components could be a potential strategy. In addition, carnosine also reduced the mRNA expression of VEGF. VEGF transcription is also regulated by Wnt/β-catenin/Tcf-4 signaling (39,40). Previous studies (44,45) have shown that tolfenamic acid, a fenamic acid-derived nonsteroid anti-inflammatory drug, can downregulate β-catenin mRNA expression in a dose- and time-dependent manner to reduce the proliferation of human colon cancer cell lines. Tolfenamic acid also decreases the expression of the β-catenin target gene VEGF, leading to reduced angiogenesis in human colon cancer cell lines (44). *Ginkgo biloba* exocarp extracts (GBEE) can suppress Wnt3a and β-catenin protein expression and VEGF mRNA levels in Lewis lung cancer (LLC) cells (45). GBEE can also inhibit the growth of LLC-transplanted tumors in C57BL/6 mice in a dose-dependent manner by suppressing tumor growth in the lungs by reducing β-catenin and VEGF protein expression (45). These studies and the present results show that carnosine can reduce cell proliferation and that angiogenesis may inhibit the Wnt/β-catenin/Tcf-4 signaling pathway. In the future, investigating the cellular Wnt expression and β-catenin-DNA binding activity may aid understanding of the effect of carnosine in cell proliferation through regulating the Wnt/catenin signaling.

In the present study, carnosine significantly increased Beclin-1, PI3K III and LC-3 mRNA levels and the level of autophagy. These results showed that carnosine reduced not only cell proliferation but also cell viability by inducing autophagy. Beclin-1 is one of three core activated autophagic complex proteins: Beclin-1, Vps34 and Bcl-2 (46). When Bcl-2 is phosphorylated, Beclin-1 is activated, followed by PI3K III and LC3-II, triggering autophagosome formation (47). Beclin-1/PI3K III/LC3-II signaling pathways are involved in preautophagosome formation (48). Therefore, the above molecules are changed by carnosine, which leads to autophagy in HCT-116 cells. A previous study shows that quercetin can induce autophagy in human gastric cells by increasing LC3-II and Beclin-1 expression (25). The clinical chemotherapy imatinib induces cellular autophagy by increasing the levels of PI3K and LC-3 and inhibiting the viability of leukemia cells (49). Previous studies (50–53) have shown that *Grias neuberthii* extract can induce autophagy in human colon cancer cells by increasing intracellular Beclin-1 and LC-3 levels (50). Our previous studies also showed that sedanolide and α-phellandrene, which are active components of celery, can increase PI3K, Beclin-1 and LC-3 protein levels, leading to autophagy induction in human colorectal HCT-116 cancer cells and liver J-5 cancer cells (51,52). Salidroside, a natural active ingredient extracted from *Rhodiola rosea*, has been shown to decrease the expression of autophagy proteins, suggesting that salidroside induces autophagy through the PI3K/Akt/mTOR pathway in human gastric cancer (53). Based on the aforementioned studies (25,49–52), the autophagic induction mechanism of carnosine is similar to the autophagic induction by quercetin, imatinib, *Grias neuberthii* extract and α-phellandrene, and this may be attributed to the induction of autophagy via the upregulation of Beak-1, PI3K and LC3 expression. However, except for the Beclin-1/PI3K III/LC3-II signaling pathways investigated in the present study, the effects of carnosine on PI3K/Akt/mTOR signaling pathway in autophagy need future investigation.

Necroptosis is another mode of programmed cell death that differs from apoptosis (54). Necroptosis is also a cell death pathway, including programmed necroptosis, coercion, iron death and mitochondrial permeability transition, which is regulated by intracellular molecules. Among them, necroptosis is regulated by RIP3 and MLKL (55). Additionally, ROS production involves the stabilization of the necrosome complex composed of RIP1 and RIP3 (56). In the present study, carnosine significantly increased ROS and MLKL levels but decreased ATP levels. These are all major regulatory molecules of necroptosis. A previous study showed that apigenin could induce necroptosis by increasing ROS levels and reducing ATP levels and MLKL phosphorylation (57). Liu *et al* (58) showed that tanshinone A, a major compound of *Salvia miltiorrhiza* Bunge (Danshen), could induce necroptosis by increasing ROS, depleting ATP and downregulating MLKL to reduce the viability of lung NCI-H1299 and A549 cells. The findings of the present study revealed that carnosine could increase the expression of ROS and MLKL but decreased ATP levels to induce necroptosis in HCT-116 cells.

HIF-1, a heterodimer that binds to hypoxia-responsive elements, is one of the major regulatory molecules involved in cancer cell proliferation and metastasis and activates VEGF transcription (59). VEGF is involved in tumor cell proliferation and the regulation of blood vessel density (60). Additionally, activated EGFR signaling leads to the proliferation of epidermal cells to induce tumor formation under hypoxic conditions (60). In the present study, carnosine significantly reduced angiogenesis by decreasing VEGF, EGFR and HIF-1α expression. Huang *et al* (61) showed that wogonin, a plant-derived flavone, can reduce the angiogenesis of human breast MCF-7 cells by degrading HIF-1α protein and reducing VEGF and EGFR expression. In human astrocytoma U251 cells, hepatoma Hep3B cells and an HUVEC culture experimental model, the oligomer procyanidin, which is isolated from grape seeds, can inhibit angiogenesis by suppressing the HIF-1α-dependent pathway (62). The results of the present study and the above studies show that if cellular VEGF, EGFR and HIF-1α expression is decreased, cells can reduce angiogenesis in various cell culture models. An investigation of the suppression of carnosine in tumorigenesis in animal models is required.

Carnosine serves an important role in inhibiting non-enzyme protein glycosylation (63,64). Glycosylation, a crucial post-translational process in protein modification, is characteristic of physiological and pathological functions (64). The tumor microenvironment produces altered glycans by glycosylation that contributes to cancer progression and aggressiveness (64). Glycosylation of tumor-cell-surface glycans is involved in enhancing transient cell cycle arrest (65), regulating autophagosome forming leading to induce autophagy (66) and degrading the extracellular matrix to activate the angiogenesis (67). The reactive glycosylation rate occurs rapidly with the lysine-histidine sequence (68). Carnosine has a glycine-histidine structure similar to the lysine-histidine sequence, but it inhibits sugar-mediated cross-linking of a specific protein (69). The tumor microenvironment produces altered glycans that contribute to cancer progression and aggressiveness. Abnormal glycosylation is widely observed in tumor angiogenesis. Hipkiss and Gaunitz (69) showed that carnosine could reduce the glycosylation then reduce cell proliferation and migration.

**Figure 6.**
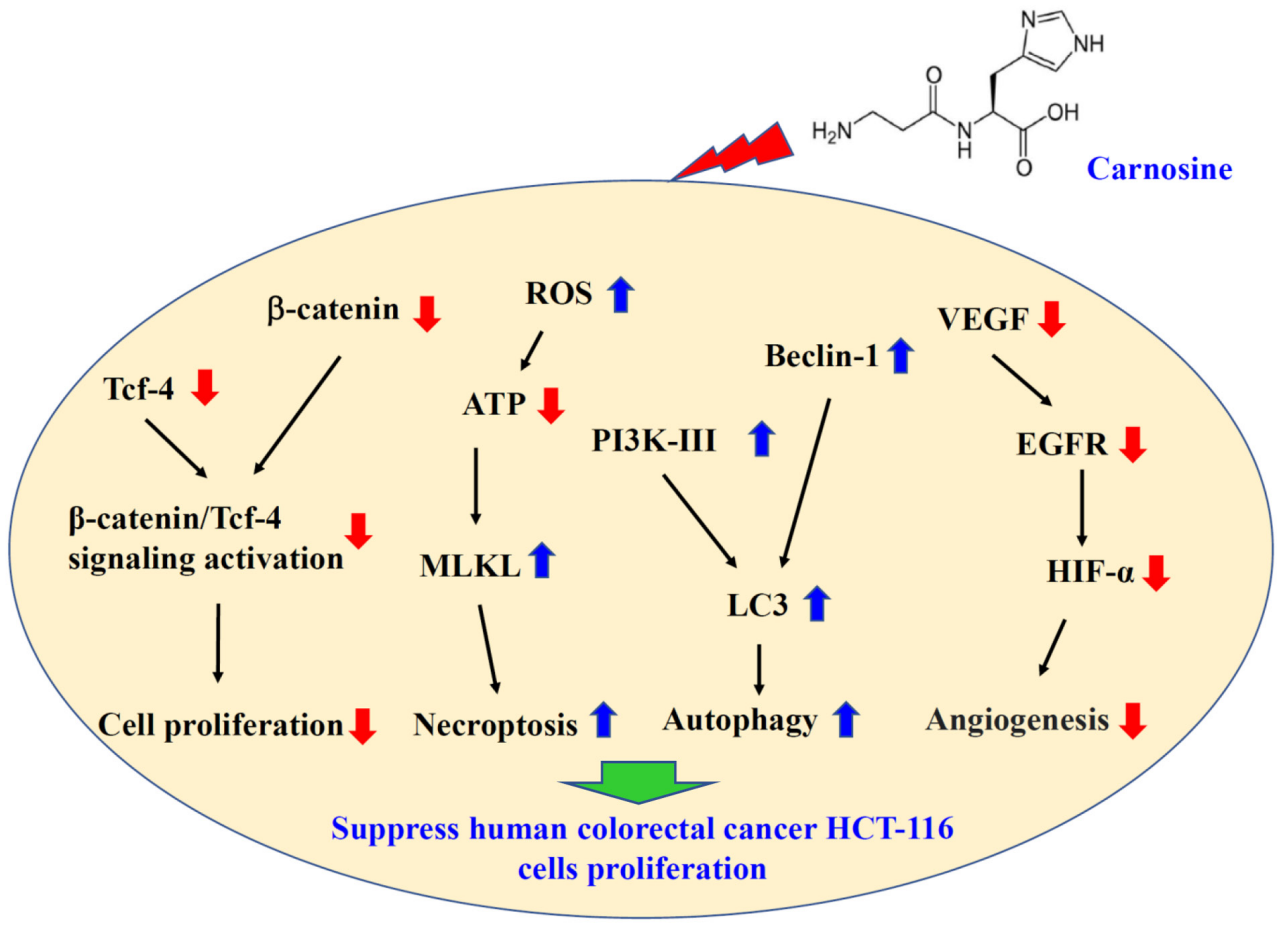
Potential mechanisms by which carnosine inhibits the proliferation of HCT-116 cells. ROS, reactive oxygen species; Tcf-4, transcription factor 4; MLKL, mixed lineage kinase domain like pseudokinase; PI3K, phosphatidylinositol 3-kinase; LC3, 1A/1B-light chain 3; VEGF, vascular endothelial growth factor; EGFR, epidermal growth factor receptor; HIF-α, hypoxia-inducible factor 1-alpha.

As shown in Fig. 6, carnosine suppressed cell proliferation by reducing β-catenin/Tcf-4 signaling activation, including inhibition of the expression of β-catenin and Tcf-4. In addition, carnosine suppressed angiogenesis by reducing VEGF, EGRF and HIF-α expression. Carnosine induced necroptosis, through reduced ATP levels and increased ROS and MLKL levels and autophagy, through increasing Beclin-1 and PI3KIII expression. In this present study, only the mRNA expression of important protein regulators involved in cell proliferation, apoptosis, autophagy and angiogenesis was analyzed but not the protein levels. There are consistent results between the mRNA expression of these molecules and the physiological functions. Li *et al* (70) show that accurate determination of mRNA levels can be used in both laboratory and clinical studies to describe the biological, pathological and clinical roles of genes in health and disease. For speedy and precise analysis of the regulatory mechanism of these regulators and cell physiological effects, mRNA analysis was used in the present study. However, the protein contents should be measured in the future; if the mRNA expression proves different from the physiological functions of these molecules it may be that some post-translation modification is involved (71).

In conclusion, the present study showed that carnosine can reduce human CRC cell viability and proliferation. Mechanistically, carnosine induced autophagy and necroptosis and reduced angiogenesis in HCT-116 cells. In the context of cancer prevention and therapy, understanding the molecular regulatory mechanisms and animal studies are required in the future.

## Figures and Tables

**Table I.** List of primer sequences used for reverse transcription-quantitative PCR analysis.

Gene	Forward sequence (5′-3′)	Reverse sequence (5′-3′)	GenBank accession no.
β-actin	ATGTGCAAGGCCGGCTTC	GAATCCTTCTGACCCATGCC	NM001101.3
Tcf-4	ACCAGCAACCAGCACTTTCC	GCCCAACATTCCTGCATAGC	NM001083962.2
Bax	TGTTTTCTGACGGCAACTTCA	AGCCCATGATGGTTCTGATCA	NM001291428.1
Bcl-2	CCTGTGGATGACTGAGTACCTGAAC	CAGCCAGGAGAAATCAAACAGA	NM000633.2
Caspase-3	TGGATTATCCTGAGATGGGTTTATG	GCTGCATCGACATCTGTACCA	NM004346.3
Caspase-8	TCCAAATGCAAACTGGATGATG	TTTTCAGGATGTCCAACTTTCCTT	NM001080124.1
PARP	TGGTCAAGACACAGACACCCA	ACGGAGGCGCTGGTTTCT	NM001618.4
MLKL	CCTGGGCACAGGAAGATCAG	TTTCTAATCGTCTCAGTGAAGCTTCT	NM001142497.2
Beclin-1	CTGGACACGAGTTTCAAGATCCT	GTTAGTCTCTTCCTCCTGGGTCTCT	NM001313998.1
LC3	TCCTGGACAAGACCAAGTTTTTG	ACCATGCTGTGCTGGTTCAC	NM032514.3
PI3K III	TTGGAGACAGGCACCTGGAT	CCATTTCTTTATTCAGCTTCATTGG	NM001308020.1
VEGF	CGAGGGCCTGGAGTGTGT	TGGTGAGGTTTGATCCGCATA	NM_001025366.2
EGFR	GGCGTCCGCAAGTGTAAGAA	TCGTAGCATTTATGGAGAGTGAGTCT	NM_005228.5
HIF-1α	TAACTTTGCTGGCCCCAGC	ACTTCCTCAAGTTGCTGGTCATC	NM_001243084.1

Tcf-4, transcription factor 4; Bax, Bcl-2-associated X protein; Bcl-2, B-cell lymphoma-2; PARP, poly(ADP-ribose) polymerase; MLKL, mixed lineage kinase domain like pseudokinase; LC3, 1A/1B-light chain 3; PI3K, phosphatidylinositol 3-kinase; VEGF, vascular endothelial growth factor; EGFR, epidermal growth factor receptor; HIF-α, hypoxia-inducible factor 1-alpha.

## Data Availability

The datasets used and/or analyzed during the present study are available from the corresponding author on reasonable request.

## Abbreviations

CRC: colorectal cancer
EGFR: epidermal growth factor receptor
HIF-α: hypoxia-inducible factor 1-alpha
LC3: 1A/1B-light chain 3
MLKL: mixed lineage kinase domain like pseudokinase
mTOR: mammalian target of rapamycin
PI3K: phosphatidylinositol 3-kinase
RIP3: receptor interaction protein 3
Tcf-4: transcription factor 4
VEGF: vascular endothelial growth factor

## References

[1] Boldyrev AA, Aldini G, Derave W (2013). Physiology and pathophysiology of carnosine. Physiol Rev.

[2] Hipkiss AR, Baye E, de Courten B (2016). Carnosine and the processes of ageing. Maturitas.

[3] Jain S, Kim ES, Kim D, Burrows D, De Felice M, Kim M, Baek SH, Ali A, Redgrave J, Doeppner TR (2020). Comparative cerebroprotective potential of d- and l-carnosine following ischemic stroke in mice. Int J Mol Sci.

[4] Prokopieva VD, Yarygina EG, Bokhan NA, Ivanova SA (2016). Use of carnosine for oxidative stress reduction in different pathologies. Oxid Med Cell Longev.

[5] Caruso G, Fresta CG, Musso N, Giambirtone M, Grasso M, Spampinato SF, Merlo S, Drago F, Lazzarino G, Sortino MA (2019). Carnosine prevents Aβ-induced oxidative stress and inflammation in microglial cells: A key role of TGF-β1. Cells.

[6] Bermúdez ML, Seroogy KB, Genter MB (2019). Evaluation of carnosine intervention in the Thy1-aSyn mouse model of Parkinson's disease. Neuroscience.

[7] Hsieh SL, Hsieh S, Lai PY, Wang JJ, Li CC, Wu CC (2019). Carnosine suppresses human colorectal cell migration and intravasation by regulating EMT and mMP expression. Am J Chin Med.

[8] Wu CC, Lai PY, Hsieh S, Cheng CC, Hsieh SL (2019). Suppression of carnosine on adhesion and extravasation of human colorectal cancer cells. Anticancer Res.

[9] Zhang Z, Miao L, Wu X, Liu G, Peng Y, Xin X, Jiao B, Kong X (2014). Carnosine inhibits the proliferation of human gastric carcinoma cells by retarding Akt/mTOR/p70S6K signaling. J Cancer.

[10] Tamaki N, Funatsuka A, Fujimoto S, Hama T (1984). The utilization of carnosine in rats fed on a histidine-free diet and its effect on the levels of tissue histidine and carnosine. J Nutr Sci Vitaminol (Tokyo).

[11] Gariballa SE, Sinclair AJ (2000). Carnosine: Physiological properties and therapeutic potential. Age Ageing.

[12] Zhao K, Li Y, Wang Z, Han N, Wang Y (2019). Carnosine protects mouse podocytes from high glucose induced apoptosis through PI3K/AKT and Nrf2 pathways. BioMed Res Int.

[13] Lee J, Park JR, Lee H, Jang S, Ryu SM, Kim H, Kim D, Jang A, Yang SR (2018). L-carnosine induces apoptosis/cell cycle arrest via suppression of NF-κB/STAT1 pathway in HCT116 colorectal cancer cells. In Vitro Cell Dev Biol Anim.

[14] Joshi RK, Kim WJ, Lee SA (2014). Association between obesity-related adipokines and colorectal cancer: A case-control study and meta-analysis. World J Gastroenterol.

[15] Bray F, Ferlay J, Soerjomataram I, Siegel RL, Torre LA, Jemal A (2018). Global cancer statistics 2018: GLOBOCAN estimates of incidence and mortality worldwide for 36 cancers in 185 countries. CA Cancer J Clin.

[16] Chaabane W, User SD, El-Gazzah M, Jaksik R, Sajjadi E, Rzeszowska-Wolny J, Los MJ (2013). Autophagy, apoptosis, mitoptosis and necrosis: Interdependence between those pathways and effects on cancer. Arch Immunol Ther Exp (Warsz).

[17] Martinez-Font E, Pérez-Capó M, Ramos R, Felipe I, Garcías C, Luna P, Terrasa J, Martín-Broto J, Vögler O, Alemany R (2020). Impact of Wnt/β-Catenin Inhibition on Cell Proliferation through CDC25A Downregulation in Soft Tissue Sarcomas. Cancers (Basel).

[18] Hikita H, Kodama T, Shimizu S, Li W, Shigekawa M, Tanaka S, Hosui A, Miyagi T, Tatsumi T, Kanto T (2012). Bak deficiency inhibits liver carcinogenesis: A causal link between apoptosis and carcinogenesis. J Hepatol.

[19] Ahamed M, Akhtar MJ, Siddiqui MA, Ahmad J, Musarrat J, Al-Khedhairy AA, AlSalhi MS, Alrokayan SA (2011). Oxidative stress mediated apoptosis induced by nickel ferrite nanoparticles in cultured A549 cells. Toxicology.

[20] Galati S, Boni C, Gerra MC, Lazzaretti M, Buschini A (2019). Autophagy: A player in response to oxidative stress and DNA damage. Oxid Med Cell Longev.

[21] Shimizu S, Yoshida T, Tsujioka M, Arakawa S (2014). Autophagic cell death and cancer. Int J Mol Sci.

[22] Robinson N, Ganesan R, Hegedűs C, Kovács K, Kufer TA, Virág L (2019). Programmed necrotic cell death of macrophages: Focus on pyroptosis, necroptosis, and parthanatos. Redox Biol.

[23] Wu W, Liu P, Li J (2012). Necroptosis: An emerging form of programmed cell death. Crit Rev Oncol Hematol.

[24] Manda G, Isvoranu G, Comanescu MV, Manea A, Debelec Butuner B, Korkmaz KS (2015). The redox biology network in cancer pathophysiology and therapeutics. Redox Biol.

[25] Wang K, Liu R, Li J, Mao J, Lei Y, Wu J, Zeng J, Zhang T, Wu H, Chen L (2011). Quercetin induces protective autophagy in gastric cancer cells: Involvement of Akt-mTOR- and hypoxia-induced factor 1α-mediated signaling. Autophagy.

[26] Fulda S (2010). Cell death and survival signaling in oncogenesis. Klin Padiatr.

[27] Denizot F, Lang R (1986). Rapid colorimetric assay for cell growth and survival. Modifications to the tetrazolium dye procedure giving improved sensitivity and reliability. J Immunol Methods.

[28] Chomczynski P, Sacchi N (1987). Single-step method of RNA isolation by acid guanidinium thiocyanate-phenol-chloroform extraction. Anal Biochem.

[29] Livak KJ, Schmittgen TD (2001). Analysis of relative gene expression data using real-time quantitative PCR and the 2(−ΔΔC(T)) Method. Methods.

[30] Cho YH, Ro EJ, Yoon JS, Mizutani T, Kang DW, Park JC, Il Kim T, Clevers H, Choi KY (2020). 5-FU promotes stemness of colorectal cancer via p53-mediated WNT/β-catenin pathway activation. Nat Commun.

[31] Kanzawa T, Germano IM, Komata T, Ito H, Kondo Y, Kondo S (2004). Role of autophagy in temozolomide-induced cytotoxicity for malignant glioma cells. Cell Death Differ.

[32] Prieto-Domínguez N, Ordóñez R, Fernández A, García-Palomo A, Muntané J, González-Gallego J, Mauriz JL (2016). Modulation of autophagy by sorafenib: Effects on treatment response. Front Pharmacol.

[33] Wu Y, Dong G, Sheng C (2020). Targeting necroptosis in anticancer therapy: Mechanisms and modulators. Acta Pharm Sin B.

[34] Capozzi M, De Divitiis C, Ottaiano A, von Arx C, Scala S, Tatangelo F, Delrio P, Tafuto S (2019). Lenvatinib, a molecule with versatile application: From preclinical evidence to future development in anti-cancer treatment. Cancer Manag Res.

[35] Chao DL, Sanchez CA, Galipeau PC, Blount PL, Paulson TG, Cowan DS, Ayub K, Odze RD, Rabinovitch PS, Reid BJ (2008). Cell proliferation, cell cycle abnormalities, and cancer outcome in patients with Barrett's esophagus: A long-term prospective study. Clin Cancer Res.

[36] Tiwari M (2011). Apoptosis and survival. Indian J Hum Genet.

[37] Loo G (2003). Redox-sensitive mechanisms of phytochemical-mediated inhibition of cancer cell proliferation (review). J Nutr Biochem.

[38] Shi Y, Zhang CJ (2011). The effects of carnosine on high glucose-induced apoptosis of human umbilical vein endothelial cells. Adv Mat Res.

[39] Mateyak MK, Obaya AJ, Sedivy JM (1999). c-Myc regulates cyclin D-Cdk4 and -Cdk6 activity but affects cell cycle progression at multiple independent points. Mol Cell Biol.

[40] Tian Y, Wan H, Tan G (2012). Cell cycle-related kinase in carcinogenesis. Oncol Lett.

[41] Sebio A, Kahn M, Lenz HJ (2014). The potential of targeting Wnt/β-catenin in colon cancer. Expert Opin Ther Targets.

[42] Sakoguchi-Okada N, Takahashi-Yanaga F, Fukada K, Shiraishi F, Taba Y, Miwa Y, Morimoto S, Iida M, Sasaguri T (2007). Celecoxib inhibits the expression of survivin via the suppression of promoter activity in human colon cancer cells. Biochem Pharmacol.

[43] Shan BE, Wang MX, Li RQ (2009). Quercetin inhibit human SW480 colon cancer growth in association with inhibition of cyclin D1 and survivin expression through Wnt/beta-catenin signaling pathway. Cancer Invest.

[44] Ha T, Lou Z, Baek SJ, Lee SH (2016). Tolfenamic acid downregulates β-catenin in colon cancer. Int Immunopharmacol.

[45] Han D, Cao C, Su Y, Wang J, Sun J, Chen H, Xu A (2016). *Ginkgo biloba* exocarp extracts inhibits angiogenesis and its effects on Wnt/β-catenin-VEGF signaling pathway in Lewis lung cancer. J Ethnopharmacol.

[46] Kang R, Zeh HJ, Lotze MT, Tang D (2011). The Beclin 1 network regulates autophagy and apoptosis. Cell Death Differ.

[47] Mizushima N (2010). Autophagy. FEBS Lett.

[48] Klionsky DJ, Emr SD (2000). Autophagy as a regulated pathway of cellular degradation. Science.

[49] Ertmer A, Huber V, Gilch S, Yoshimori T, Erfle V, Duyster J, Elsässer HP, Schätzl HM (2007). The anticancer drug imatinib induces cellular autophagy. Leukemia.

[50] Guamán-Ortiz LM, Romero-Benavides JC, Suarez AI, Torres-Aguilar S, Castillo-Veintimilla P, Samaniego-Romero J, Ortiz-Diaz K, Bailon-Moscoso N (2020). Cytotoxic property of *Grias neuberthii* extract on human colon cancer cells: A crucial role of autophagy. Evid Based Complement Alternat Med.

[51] Hsieh LC, Hsieh SL, Chen CT, Chung JG, Wang JJ, Wu CC (2015). Induction of α-phellandrene on autophagy in human liver tumor cells. Am J Chin Med.

[52] Hsieh SL, Chen CT, Wang JJ, Kuo YH, Li CC, Hsieh LC, Wu CC (2015). Sedanolide induces autophagy through the PI3K, p53 and NF-κB signaling pathways in human liver cancer cells. Int J Oncol.

[53] Rong L, Li Z, Leng X, Li H, Ma Y, Chen Y, Song F (2020). Salidroside induces apoptosis and protective autophagy in human gastric cancer AGS cells through the PI3K/Akt/mTOR pathway. Biomed Pharmacother.

[54] Galluzzi L, Kepp O, Chan FK, Kroemer G (2017). Necroptosis: Mechanisms and relevance to disease. Annu Rev Pathol.

[55] Dhuriya YK, Sharma D (2018). Necroptosis: A regulated inflammatory mode of cell death. J Neuroinflammation.

[56] Schenk B, Fulda S (2015). Reactive oxygen species regulate Smac mimetic/TNFα-induced necroptotic signaling and cell death. Oncogene.

[57] Lee YJ, Park KS, Nam HS, Cho MK, Lee SH (2020). Apigenin causes necroptosis by inducing ROS accumulation, mitochondrial dysfunction, and ATP depletion in malignant mesothelioma cells. Korean J Physiol Pharmacol.

[58] Liu X, Zhang Y, Gao H, Hou Y, Lu JJ, Feng Y, Xu Q, Liu B, Chen X (2020). Induction of an MLKL mediated non-canonical necroptosis through reactive oxygen species by tanshinol A in lung cancer cells. Biochem Pharmacol.

[59] Jackson AL, Zhou B, Kim WY (2010). HIF, hypoxia and the role of angiogenesis in non-small cell lung cancer. Expert Opin Ther Targets.

[60] Lichtenberger BM, Tan PK, Niederleithner H, Ferrara N, Petzelbauer P, Sibilia M (2010). Autocrine VEGF signaling synergizes with EGFR in tumor cells to promote epithelial cancer development. Cell.

[61] Huang KF, Zhang GD, Huang YQ, Diao Y (2012). Wogonin induces apoptosis and down-regulates survivin in human breast cancer MCF-7 cells by modulating PI3K-AKT pathway. Int Immunopharmacol.

[62] Zheng HL, Yang J, Hou Y, Sun B, Zhang Q, Mou Y, Wand L, Wu C (2015). Oligomer procyanidins (F2) isolated from grape seeds inhibits tumor angiogenesis and cell invasion by targeting HIF-1α *in vitro*. Int J Oncol.

[63] Quinn PJ, Boldyrev AA, Formazuyk VE (1992). Carnosine: Its properties, functions and potential therapeutic applications. Mol Aspects Med.

[64] Hipkiss AR (1998). Carnosine, a protective, anti-ageing peptide?. Int J Biochem Cell Biol.

[65] Sampathkumar SG, Jones MB, Meledeo MA, Campbell CT, Choi SS, Hida K, Gomutputra P, Sheh A, Gilmartin T, Head SR (2006). Targeting glycosylation pathways and the cell cycle: Sugar-dependent activity of butyrate-carbohydrate cancer prodrugs. Chem Biol.

[66] Fahie K, Zachara NE (2016). Molecular functions of glycoconjugates in autophagy. J Mol Biol.

[67] Cheng WK, Oon CE (2018). How glycosylation aids tumor angiogenesis: An updated review. Biomed Pharmacother.

[68] Hipkiss AR, Michaelis J, Syrris P (1995). Non-enzymatic glycosylation of the dipeptide L-carnosine, a potential anti-protein-cross-linking agent. FEBS Lett.

[69] Hipkiss AR, Gaunitz F (2014). Inhibition of tumour cell growth by carnosine: some possible mechanisms. Amino Acids.

[70] Li Y, Wang K, Chen L, Zhu X, Zhou J (2016). Quantification of mRNA Levels Using Real-Time Polymerase Chain Reaction (PCR). Methods Mol Biol.

[71] Deribe YL, Pawson T, Dikic I (2010). Post-translational modifications in signal integration. Nat Struct Mol Biol.

